# Relationship Between Physical Activity Level and Primary Care Costs in Older Diabetic Patients From a Middle-Size Brazilian City: An Eight-Year Follow-Up Study

**DOI:** 10.3389/ijph.2025.1607605

**Published:** 2025-01-29

**Authors:** Kelly Akemi Kikuti Koyama, Monique Yndawe Castanho Araujo, Luana Carolina de Morais, Italo Ribeiro Lemes, Rômulo Araújo Fernandes, Bruna Camilo Turi-Lynch, Flávia Mori Sarti, Thais Cristina Delacosta, Henrique Luiz Monteiro, Jamile Sanches Codogno

**Affiliations:** ^1^ Laboratory of Investigation in Exercise (LIVE), Department of Physical Education, Sao Paulo State University -UNESP, Sao Paulo, Brazil; ^2^ Department of Physical Therapy, Sao Paulo State University -UNESP, Sao Paulo, Brazil; ^3^ Physical Education and Exercise Science Department, Lander University, Greenwood, SC, United States; ^4^ University of Sao Paulo, USP East, School of Arts, Sciences and Humanities, São Paulo, Brazil; ^5^ Department of Physical Education, Sao Paulo State University -UNESP, Bauru, Sao Paulo, Brazil

**Keywords:** diabetes mellitus, physical activity, primary care, health care costs, epidemiology

## Abstract

**Objectives:**

Physical activity and costs have been consistently related each other, but mostly in cross-sectional investigations. This study aims to investigate the relationship between changes in physical activity level and changes in healthcare costs among older diabetic adults in an 8-year follow-up study.

**Methods:**

The study followed 151 diabetic adults ≥50 years of age, for a period of 8 years, who were patients of Basic Health Care Units in the city of Bauru (Brazil). Medical records were consulted to obtain information on healthcare costs. Physical activity level was assessed through an interview. Data analysis included descriptive statistics, analysis of variance, and linear regression.

**Results:**

Participants who increased leisure-time physical activity from 2010 to 2018 accumulated less healthcare costs from 2020 to 2018. The magnitude of the relationship was small (r = −0.233 [95% CI: −0.379 to −0.076]).

**Conclusion:**

In summary, among diabetic patients, to increase leisure-time physical activity from 2010 to 2018 was inversely related to the amount of healthcare costs spent over the same period of 8 years.

## Introduction

Diabetes mellitus (DM) is a clinical condition characterized by a deficiency in the action/secretion of insulin, leading to abnormal alterations in blood glucose levels [1]. Over the last decades, concerns about the social and economic impact of DM have grown due to the relevant number of people living with DM worldwide (536.6 million), and figures show an increasing trend in both its prevalence [1] and related comorbidities [1–3]. In terms of economic burden, DM is a clinical condition related to substantial economic losses in healthcare services [2, 3], especially because DM is linked to obesity and other comorbidities [4].

In terms of the prevention and treatment of DM, the consistent relationship between obesity and DM makes the role of physical activity as a non-pharmacological tool relevant to its prevention/treatment [4, 5]. Moreover, the beneficial impact of physical activity level on economic outcomes has been previously described [6, 7], in which higher physical activity level has been linked to lower productivity losses, lower costs attributed to medicine use, and lower utilization of healthcare services [7–11].

In fact, health organizations support the use of physical activity as a tool in the prevention and treatment of DM, especially because the positive impact of regular physical activity on increased insulin sensitivity and weight loss/maintenance reduces the risk of diabetes-related complications and cardiovascular risk factors (e.g., high blood pressure and dyslipidemias) [12]. On the other hand, the potential impact of physical activity on mitigation of healthcare costs among diabetic patients is still under investigation [5, 13, 14]. In the general population (non-diabetic adults), it has been suggested that higher physical activity levels may be able to mitigate ∼1% of all healthcare costs [13]. Among diabetic patients, it is suggested that higher physical activity can reduce the costs attributed to medical consultations and medicines for the treatment of comorbidities [14], but most of the evidence available is supported by cross-sectional data from developed nations, limiting both the determination of causal relationships [14] and inferences of such findings to developing nations (settings where physical activity and the access to health services are substantially different from Europe, United States, Canada and Australia).

Therefore, considering the importance of physical activity in healthcare services (as an accessible non-pharmacological approach to promote health in both developed and developing nations), the aim of the current study was to investigate the relationship between changes in physical activity level and healthcare costs among older diabetic adults living in a middles-size Brazilian city.

## Methods

### Sample Design and Selection

The present study presented a descriptive research model, with longitudinal analysis. Baseline measures were carried out in 2010 and all participants were interviewed/assessed every 2 years (2010, 2012, 2014, 2018, and 2018). Before every survey, the research team contacted the participants or family members by phone to schedule the interview and to identify any death. For those participants who agreed to participate, interviews were carried out by trained researchers who followed a structured interview with the same questionnaires used from 2010 to 2018 (e.g., anthropometric measures, sociodemographic, smoking and physical activity questionnaires).

Participants were recruited in primary care facilities of the Brazilian National Health Service (BNHS) in the city of Bauru, State of Sao Paulo. Bauru is a medium-size city (∼360,000 inhabitants; Human Development Index [HDI] = 0.801) located in the central region of the State of Sao Paulo, Brazil. The study was approved by the Ethics Committee of the xxxxxxxxxx (xxxxx), xxxxx, xx, xxxxx (xxxxxxx/xxxxxxxxxxx), and all participants signed the written consent forms.

Healthcare services provided by the BNHS are free of charge to the population. The BNHS primary care is composed of Basic Healthcare Units (BHU), which are facilities offering low complexity health procedures (e.g., medical consultations, dentistry, nursing, vaccination), and health promotion and prevention activities within a specific region of the municipality. The BHUs are spread out around the entire city and, considering the metropolitan area of the city, the largest BHU in each geographical region of the city (North, South, East, West, and Central) was selected to participate in the baseline measures of the study in 2010. Inclusion criteria were: 1) age ≥50 years; 2) at least one medical consultation in the BHU within the last 6 months; and 3) agree to participate in the study and sign the informed consent form.

The sample size was calculated based on the percentage of the population exclusively relying on healthcare provided by the BNHS (60%), a standard error of 3.8%, significance of 5% (z = 1.96), and design effect of 50%; resulting in 960 participants (192 in each BHU). After recruitment, 970 patients were randomly selected and completed all measurements proposed at baseline (194 in each BHU).

The initial number of diabetics in the sample was 276, but 47 of them had some missing data in any of the variables considered (the final sample and those participants excluded were similar in terms of age, sex, economic condition, smoking and waist circumference). Moreover, 78 participants started the follow-up period as non-diabetic, and they became diabetic from 2010 to 2018 (those participants also were excluded). Thus, the analysis presented in this study refers to a sub-sample of 151 individuals who were diagnosed with DM since 2010 (medical diagnosis in the medical record) and who had no missing data from 2010 to 2018.

### Sample Characterization Variables

Sociodemographic variables, including sex, age, ethnicity, and economic condition (EC) were assessed by a sociodemographic questionnaire. EC was assessed through a standard questionnaire from the Brazilian Association of Research Companies [15] and participants were categorized according to five strata of average household income (classes A [highest] to E [lowest]). Body weight (kg) and height (m) were measured using a digital scale and wall stadiometer, respectively. Information on weight and height were used to calculate the body mass index (BMI), categorized into 1) eutrophic (BMI 18.5–24.9 kg/m^2^); 2) overweight (BMI 25–29.9 kg/m^2^); and 3) obesity (BMI ≥30 kg/m^2^) [10]. Waist circumference (WC) was verified following the protocol of Lohman et al. [16].

A structured questionnaire with dichotomous question (yes or no) was applied to assess tobacco use. Furthermore, at the time of the interviews, patients also reported, through pre-established questions with dichotomic possibility of answering the presence or absence of comorbidities: hypertension, dyslipidemia, stroke, osteoporosis, and low back pain (this information was confirmed in the medical records of the participant).

### Dependent Variable: Healthcare Costs

The monetary values were estimated from the perspective of the BNHS, using micro-costing approaches (bottom up) to estimate costs. Data about direct healthcare costs were assessed through medical records, in a time horizon of 8 years. All primary care services offered by the BNHS are registered in the patient medical record (e.g., consultations [including the description of medical specialties], and medicines discharged [type and quantity]). During the years of the research, information from the medical records began to be entered into BNHS digital databases, therefore, the research team obtained authorization from the Municipal Secretary of Health to access both the patient’s physical (stored at the UBS) and digital medical records [14, 17]. The following data were extracted from the medical records: 1) number of medical and screening consultations; 2) medications (general medication); and 3) laboratory tests.

The Municipal Secretary of Health provided the amount paid by each service [14, 17]. For the analyses, the total healthcare costs (including items i, ii, and iii) and healthcare costs directly attributed to DM (DM-related medication, i.e., insulin and DM oral medication) were considered. All costs were adjusted according to the inflation observed in the Brazilian economy (Extended National Consumer Price Index [IPCA, in Brazilian Portuguese]), considering the period of August 2010 to August 2020. After adjustment by inflation, healthcare costs were converted to U.S. dollars (US$), according to the Brazilian Central Bank in September 2020 (https://www.bcb.gov.br/).

### Independent Variable: Physical Activity Level

Physical activity level (PAL) was assessed through face-to-face (2010, 2012 and 2016) and phone (2014 and 2018) interviews using the Baecke questionnaire [18], previously validated for Brazilian Portuguese and recommended for epidemiological studies [19]. The structured questionnaire assesses PAL in three domains: occupational (8 questions), sports (4 questions), and leisure-time (4 questions). Using a scale of 1–5 (Likert scale), the frequency of activities in each domain is verified (ranging from never to always).

In the occupational section of the questionnaire, there are questions about time spent sitting, standing, walking and carrying weights during labor activities. In addition, the individual also reports on fatigue and whether they consider their work to be heavier or lighter than people of the same age. This domain considers domestic activities if the individual either is retired or has no other labor occupation. In the domain of sports activities, the questionnaire considers intensity, frequency and daily time spent in sports, as well as the previous time of engagement in such activity. Finally, in the domain of leisure activities and transportation, the questionnaire considers the frequency of activities such as watching television, walking and cycling.

Each domain generates a specific physical activity score, and the sum of all domains represents the PAL. The scores are established using a formula proposed in the Baecke questionnaire [18]. The possible PAL score ranges from 3 to 15 (higher scores denote higher physical activity). In this manuscript, the change (score in 2018 minus the score and 2010) for total physical activity level score, occupational score, sports score and leisure-time score were considered.

### Statistical Analysis

**FIGURE 1:**
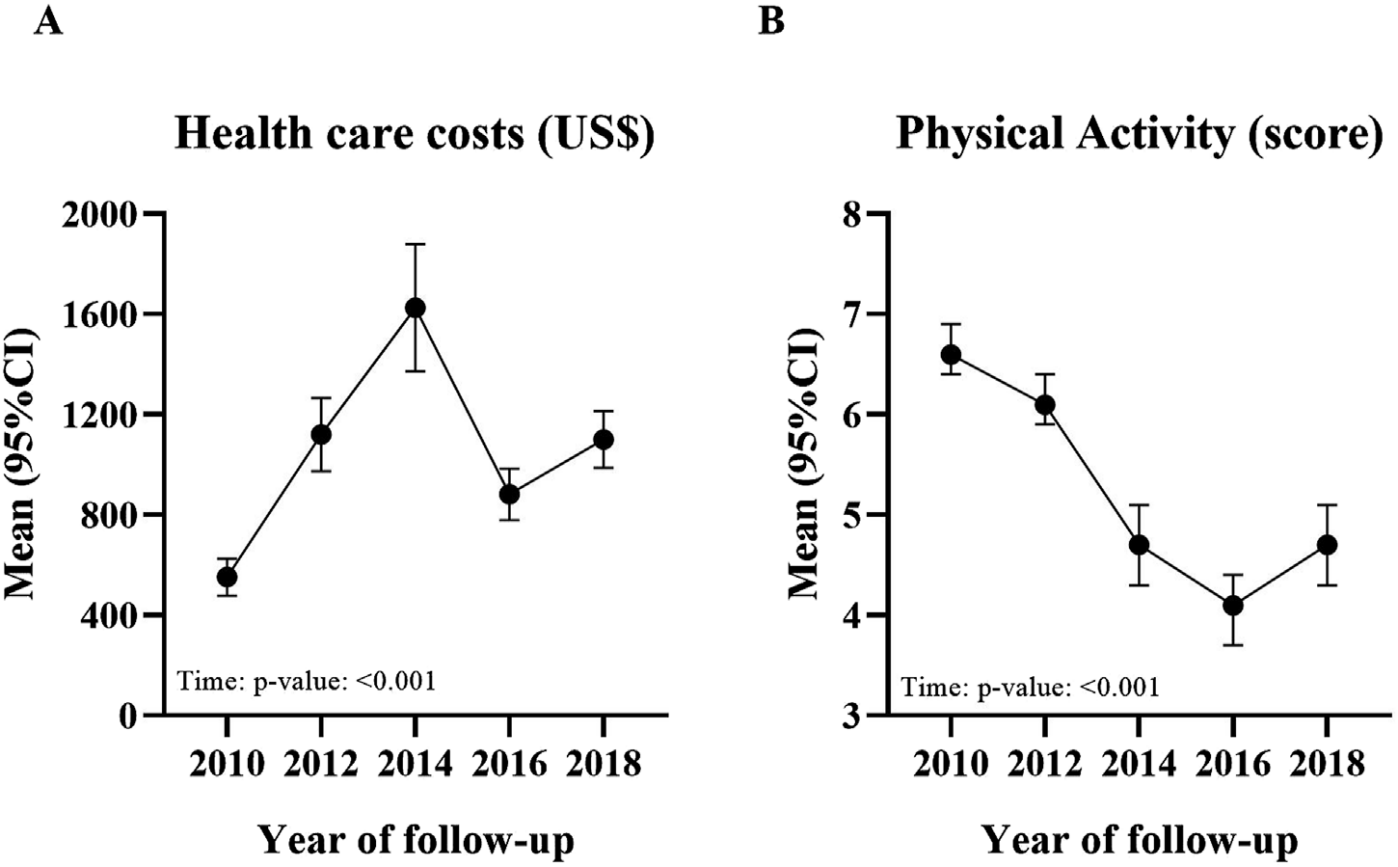
Trajectory of healthcare costs **(A)** and physical activity level **(B)** in diabetic patients from 2010 to 2018 (Bauru, Brazil. 2020).

In terms of descriptive statistics, continuous variables are presented as mean, standard deviation, median and interquartile range, depending on the distribution. Comparisons between groups [Men versus Women (Table 1)] were performed using the Student’s t-test (numerical variables) and the chi-square test (categorical variables). Comparisons of mean values [health expenditures (Figure 1A) and PA levels (Figure 1B)] across the follow-up period were based on analysis of variance (ANOVA) for repeated measures (when ANOVA was statistically significant, the Bonferroni *post hoc* test was used). The Mauchly test of sphericity was used to assess how well the models were fitted, and when necessary (sphericity assumption violated), the Greenhouse–Geisser correction was used. ANOVA for repeated measures provided measures of effect-size expressed as eta-squared values, which was classified as small (≤0.060), moderate (from 0.061 to 0.139) and large effect-size (≥0.140) [20]. The relationship between changes in physical activity (score in 2018 – score in 2010) and healthcare costs accumulated from 2010 to 2018 was assessed using linear regression (expressed as standardized coefficients [*r*] and its 95% CI), while the models were adjusted by sex, age, ethnicity, waist circumference, economic condition, smoking and occupational physical activity (Table 2). Effect-size was graded was small (from *r* = 0.100 to *r* = 0.299), moderate (from *r* = 0.300 to *r* = 0.499) and large effect-size (*r* ≥ 0.500) [20]. All analyses were performed using Stata Statistical Software (Release 16; StataCorp LP, College Station, TX), and the significance level was set at p-value < 0.05.

**TABLE 1:** Characteristics of the diabetic participants at baseline according to sex (n = 151 [Bauru, Brazil. 2020]).

Variables	Men (n = 46)	Women (n = 105)	*p*-value
	Mean (SD)	Mean (SD)	
Age (years)	67.3 (9.2)	64.4 (7.8)	0.046
Body weight (kg)	79.6 (13.7)	75.6 (15.7)	0.131
BMI (kg/m^2^)	28.4 (3.7)	31.7 (6.4)	<0.001
WC (cm)	103.4 (11.5)	104.3 (13.1)	0.692
EC (score)	20.5 (5.1)	18.4 (5.9)	0.035
Physical activity (score)			
Occupational	1.5 (1.1)	2.9 (0.8)	<0.001
Sport	1.5 (0.4)	1.4 (0.2)	0.034
Leisure-time	2.7 (0.8)	2.8 (0.7)	0.755
Total	5.8 (1.3)	7.1 (1.3)	<0.001
Categorical	N (%)	N (%)	
Smoking _current_	04 (8.7%)	09 (8.6%)	1.000
Obesity _BMI≥30 kg/m_^2^	15 (32.6%)	60 (57.1%)	0.009
Hypertension _yes_	34 (73.9%)	86 (81.9%)	0.368
Dyslipidemia _yes_	13 (28.3%)	41 (39.1%)	0.276
Stroke _yes_	03 (6.5%)	05 (4.8%)	0.960
Osteoporosis _yes_	02 (4.3%)	28 (26.7%)	0.003
Low back pain _yes_	16 (34.8%)	68 (64.8%)	<0.001
Ethnicity (N [%])			0.299
White	49 (46.7%)	16 (34.8%)	
Black	24 (22.9%)	13 (28.3%)	
Asian	01 (1.0%)	01 (2.2%)	
Other	31 (29.5%)	16 (34.8%)	

DM, diabetes mellitus; SD, standard-deviation; BMI, body mass index; WC, waist circumference; EC, economic condition; PAL, physical activity level.

**TABLE 2:** Relationship between physical activity domains (difference between baseline [2010] and follow-up [2018]]) and healthcare costs accumulated from 2010 to 2018 in diabetic patients (n = 151 [Bauru, Brazil. 2020]).

	Linear regression		
	Outcome: Healthcare costs from 2010 to 2018		
	*r* (95% CI)	*p*-value	r^2^ of the model
Model – 1*			
Occupational	0.121 (−0.039–0.275)	0.178	0.048
Model – 2**			
Sport	−0.067 (−0.224 to 0.094)	0.493	0.051
Model – 3***			
Leisure-time	−0.233 (−0.379 to −0.076)	0.008	0.095
Model – 4****			
Total physical activity	−0.075 (−0.232 to 0.086)	0.441	0.033

* = linear regression adjusted by sex, age, ethnicity, waist circumference, economic status and smoking (variance inflation factor [collinearity] ranging from 1.049 to 1.351).

** linear regression adjusted by sex, age, ethnicity, waist circumference, economic status, smoking and occupational physical activity (variance inflation factor [collinearity] ranging from 1.052 to 1.398).

*** linear regression adjusted by sex, age, ethnicity, waist circumference, economic status, smoking and occupational physical activity (variance inflation factor [collinearity] ranging from 1.057 to 1.352).

**** linear regression adjusted by sex, age, ethnicity, waist circumference, economic status and smoking (variance inflation factor [collinearity] ranging from 1.053 to 1.457).

## Results

The study sample encompassed 151 participants diagnosed with DM, being 46 (30.4%) males and 105 (69.6%) females. From 2010 to 2018, these patients spent U$ 137,474.36 on primary care services in BNHS. Men and women differed in terms of age (*p*-value = 0.046), BMI (*p*-value < 0.001), economic status (*p*-value = 0.035), occupational physical activity (*p*-value < 0.001) and sports participation (*p*-value = 0.034) (Table 1).

Healthcare costs increased significantly from 2010 to 2018 [ANOVA-Time: p-value < 0.001 (Figure 1A)] in an elevated magnitude (eta-squared = 0.195). On the other hand, physical activity decreased substantially during the follow-up period [ANOVA-Time: p-value < 0.001 (Figure 1B)], also in an elevated magnitude (eta-squared = 0.287).

Changes in physical activity were not related to the amount of healthcare costs accumulated from 2010 to 2018 when considering physical activity domains: occupational and sports participation. On the other hand, participants who increased leisure-time physical activity from 2010 to 2018 accumulated less healthcare costs from 2020 to 2018. The magnitude of the relationship was small (r = −0.233 [95% CI: −0.379 to −0.076]), while the model explained poorly the outcome (9.5%).

## Discussion

This longitudinal study investigated the impact of physical activity on healthcare costs of diabetic patients. Our findings suggest that leisure-time physical activity and healthcare costs were inversely related to each other.

In terms of economic data, annual healthcare costs increased ∼20% from 2010 to 2018 in our sample. This finding was expected because it is similar to that observed in previous studies, showing an increasing trend in healthcare costs among older adults as they get older [8, 21]. In particular, scientific literature points out that this increasing trend in healthcare costs seems to be driven, at least in part, by higher demand for medicines [21]. In terms of physical activity, the physical activity score presented a different trajectory to the healthcare costs, in which there was a significant reduction from 2010 to 2018 (−25%). Although the determinants of physical activity level can vary significantly depending on the setting where this behavior is analyzed, there is scientific evidence supporting a substantial reduction in physical activity level among older adults worldwide [22].

Participants who increased leisure-time physical activity spent less healthcare costs from 2020 to 2018. The inverse relationship between physical activity and primary care costs has been shown by previous studies [23–25]. In fact, the direct impact of regular physical activity/exercise on the improvement in clinical aspects of diabetes mellitus has been evidenced by previous studies, in which different pathways are described (e.g., reduction in inflammation, insulin resistance, adiposity) [4]. The consistent interrelationship between physical activity, adiposity, and glucose metabolism seems relevant to justify the inverse relation observed between leisure-time physical activity and healthcare costs in our study. In fact, obesity is a factor with a relevant role in the economic burden attributed to DM [26, 27] and, hence, the recognized impact of physical activity on weight loss has the potential to mitigate healthcare costs in diabetic patients.

On the other hand, the relationship observed between leisure-time physical activity and healthcare costs was of small magnitude and the model poorly explained healthcare costs, denoting the participation of other variables in such phenomenon. Based on the aforementioned, some hypotheses were formulated to justify this finding. First, the impact of physical activity on healthcare costs depends on the type of service. A recent study showed that physically active individuals require less hospitalization, emergencies, and home care, while not necessarily influencing preventive services (e.g., laboratory tests, medical consultations on primary care) [23]. Active individuals probably have more awareness of their health status, and therefore may demand preventive services regularly, so that the regular practice of physical activity might be related to higher demand for preventive and primary care services [23].

In addition, DM is a chronic disease, requiring constant monitoring and, hence, costs of medication and consultations are hardly mitigated, even in sufficiently active participants. In fact, individuals with DM spent more in relation to the use of medicines for glucose control and other comorbidities (36% of direct medical costs are spent on non-diabetes-related medication and 13% on anti-hyperglycemic drugs) [28], while these expenses tend to increase with aging [21, 29]. Finally, obesity is one of the main determinants of healthcare costs among adults and a key aspect in the impact of physical activity on the mitigation of healthcare costs (through weight loss) [26, 27]. In our study body adiposity markers were related to healthcare costs, so these variables might explain (at least partially) the poor performance of the multivariate models created.

Limitations should be recognized. First, the main limitation of the study refers to the sample size. We adopted a subsample of a longitudinal study (only diabetics), which considerably decreased the number of participants in the assessment. Second, our figures represent the interaction between physical activity level and primary care costs, but no inference can be made on other economic outcomes, such as productivity losses, and secondary and tertiary care [10]. Third, physical activity level was measured by self-report, which is prone to bias recall and does not provide measures of intensity, a relevant confounder in the relation between physical activity and costs [30]. Fourth, we need to recognize that the age range of the sample collected concentrates retired individuals or those who retired during the follow-up. Therefore, although the instrument used to verify physical activity considers occupational activities in the case of retirement, in the occupational domain, the change in physical activity over time may be partially the result of the change to retirement. Finally, the higher number of women than men is a common characteristic in the primary care of the BNHS, but the reduced number of men in our sample should be considered a limitation. On the other hand, the manuscript has strong aspects. The first is its longitudinal design, which covers a period of 8 years. The second is its pragmatic characteristic (with strong external validity of the findings) as we included real world data from NHS.

In summary, among diabetic patients, the increase in leisure-time physical activity from 2010 to 2018 was inversely related to the amount of healthcare cots spent over the same period of 8 years.
